# A video corner in Facts, Views and Vision

**Published:** 2020-03-27

**Authors:** Ombelet Willem

The idea of creating a video corner has been discussed and approved by the board of our Journal.

From the beginning in 2009 Facts, Views and Vision always showed a major interest in images, art contributions, innovative techniques and visionary ideas. Therefore, we believe that this Journal provides the perfect scientific environment and conditions to initiate and support the publication of peer-reviewed scientific video articles.

It is our aim to facilitate further learning and understanding, especially when complex matters are involved. High definition audiovideo presentations, accompanied by clear and evidence based articles or case-reports provide, in our opinion, the best way of showing and explaining complicated cases and pathologies that normally require extensive writing to be described and completely understood.

This Journal embraces all ObGyn disciplines and within the context of a video corner, educational skills can be improved and learning curves can be shortened.

The management of obstetrical complications and its treatment, setting up unusual projects, the management of complications during surgical and medical treatments, new operative techniques and instrumentation, important details of an operation, rare pathologies, anatomical deviations etc. can be demonstrated in a better way and will surely have an added value in the transfer of medical information and knowledge, which would not be possible without a video presentation.

From a didactic point of view, video articles will undoubtedly be useful in training youngsters and trainees. Above this,video presentations can be stored to constitute a library dealing with challenging surgical techniques, original situations and extraordinary clinical cases.

The guidelines for authors will be very similar to that of written format articles but with restricted word counts, a few images and entries will be accompanied by a video clip. Each video article has to describe the learning objectives in a constructive and clear way and to discuss crucial scientific matters and conclusive remarks.

The video articles will be reviewed by a group of experts of each discipline in ObGyn. In the next issue of Facts, Views and Vision we will publish a few examples of a video article. From March 2020 onwards it will be possible to submit your video contributions to the Journal.

Willem Ombelet, Editor-in-Chief

